# Drug Nanocrystals in Oral Absorption: Factors That Influence Pharmacokinetics

**DOI:** 10.3390/pharmaceutics16091141

**Published:** 2024-08-29

**Authors:** Luiza de Oliveira Macedo, Jéssica Fagionato Masiero, Nádia Araci Bou-Chacra

**Affiliations:** Faculty of Pharmaceutical Sciences, University of São Paulo, Sao Paulo 05508-000, SP, Brazil

**Keywords:** drug nanocrystal, nanosuspension, oral administration, bioavailability, absorption, pharmacokinetics, dissolution rate, food effect, mucus layer, permeation

## Abstract

Despite the safety and convenience of oral administration, poorly water-soluble drugs compromise absorption and bioavailability. These drugs can exhibit low dissolution rates, variability between fed and fasted states, difficulty permeating the mucus layer, and P-glycoprotein efflux. Drug nanocrystals offer a promising strategy to address these challenges. This review focuses on the opportunities to develop orally administered nanocrystals based on pharmacokinetic outcomes. The impacts of the drug particle size, morphology, dissolution rate, crystalline state on oral bioavailability are discussed. The potential of the improved dissolution rate to eliminate food effects during absorption is also addressed. This review also explores whether permeation or dissolution drives nanocrystal absorption. Additionally, it addresses the functional roles of stabilizers. Drug nanocrystals may result in prolonged concentrations in the bloodstream in some cases. Therefore, nanocrystals represent a promising strategy to overcome the challenges of poorly water-soluble drugs, thus encouraging further investigation into unclear mechanisms during oral administration.

## 1. Introduction

Oral administration is the most common therapeutic route due to its convenience, safety, patient compliance, and cost-effectiveness [1]. Despite these advantages, orally administered drugs face challenges that compromise absorption and bioavailability, such as poor water solubility. Estimates indicate that 40% of approved drugs have low water solubility, while 90% of new chemical entities in development also exhibit this property [2]. According to the Biopharmaceutical Classification System (BCS), drug aqueous solubility and membrane permeability are key factors that influence drug bioavailability. Poorly water-soluble drugs may have high membrane permeability (BCS class II) or low membrane permeability (BCS class IV), with the limited dissolution rate being a challenge for both BCS classes during oral absorption [2].

After the oral administration of a poorly water-soluble drug, the first obstacle to overcome is its solubilization in the aqueous gastrointestinal environment. Biliary secretions, which are mainly composed of bile acids, phospholipids, and cholesterol, promote drug solubilization by incorporating lipophilic drugs into mixed micelles. Once solubilized, the drug is transported through the mucus layer and reaches the enterocyte membrane for absorption [3]. However, the short transit time in the small intestine can cause the drug to reach the colon before solubilization is complete, thus resulting in low oral bioavailability [4].

**Figure 1:**
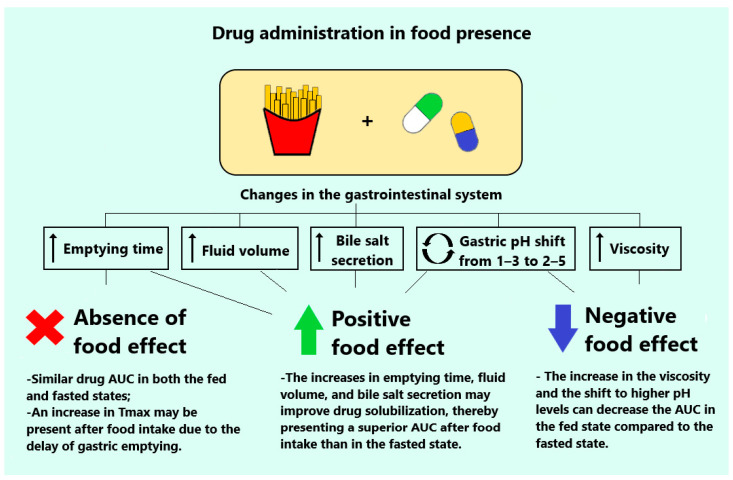
Variation in oral absorption during fed and fasted states can significantly impact the drug bioavailability. The term “absence of a food effect” denotes that drug plasma concentrations remain consistent irrespective of the individual’s fed or fasted state. In contrast, a “positive food effect” is evident when the plasma concentrations of orally administered drugs increase in the presence of food. Conversely, a “negative food effect” is characterized by a reduction in drug exposure when food is consumed.

Additionally, oral administration is susceptible to variability between the fed and fasted states (Figure 1). When a drug’s plasma concentration decreases during food intake, it is referred to as a negative food effect. Conversely, when absorption increases under these conditions, it is known as a positive food effect [5]. The presence of food in the gastrointestinal environment increases the pH, fluid volume, and motility, and delays gastrointestinal emptying [6]. While lipophilic drugs often show improved solubility and absorption in the fed state, the pH shift due to food can alter the solubility and dissolution of ionizable compounds [6,7]. Consequently, the erratic drug absorption caused by variations between the fed and fasted states can compromise patient safety, especially for drugs with multiple dosing frequencies or a low therapeutic index, which leads to poor therapeutic effects or acute/chronic toxicity [8].

Another barrier during oral administration is the mucus layer, which is a viscoelastic gel with a high water content and is mainly composed of structural glycoproteins called mucins [9]. While the mucus layer protects the intestinal epithelial surface from harmful substances and pathogens, its aqueous composition, high viscosity, and pore size of 50–1800 nm hinder the penetration of micronized poorly water-soluble drugs [3,9,10]. After traversing the mucus layer, these drugs encounter the unstirred water layer (UWL), which lies adjacent to the intestinal membrane and has a thickness of about 300 µm [11]. The hydrophilicity and thickness of the UWL present a significant challenge during the oral administration of poorly water-soluble drugs, especially for BCS class II drugs. These drug substances have a high membrane permeability, which makes UWL permeation a critical limiting step [4,11].

Once the drug has crossed the UWL, certain efflux transporters located at the enterocyte apical membrane can further compromise intestinal drug absorption. P-glycoprotein (P-gp), which is also known as multidrug resistance protein 1 (MDR1), is a well-known efflux transporter found in various cells, where it functions to limit systemic exposure to toxic compounds [12]. Both toxic compounds and orally delivered drugs that are likely P-gp substrates share lipophilicity as a common physicochemical property [13]. In the intestine, P-gp transports the drug from the apical membrane back into the intestinal lumen, thereby affecting drug absorption and distribution.

**Figure 2:**
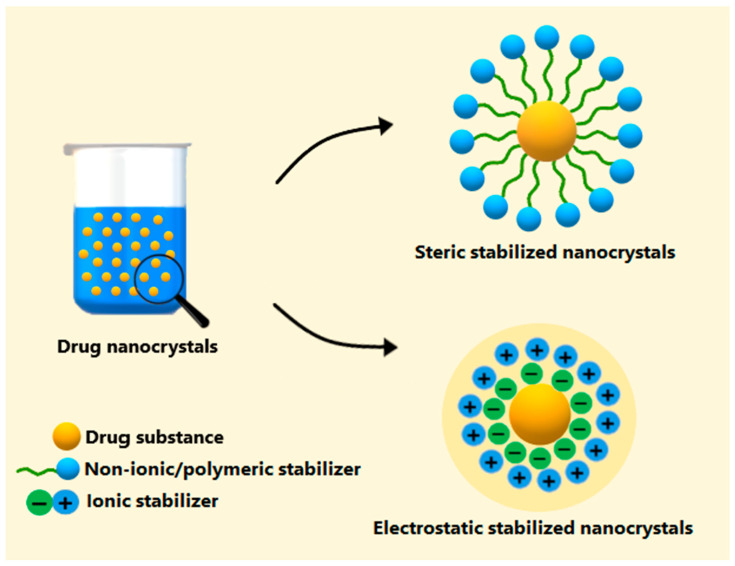
Representation of the structure of drug nanocrystals in an aqueous medium. The hydrophobic chain of a steric stabilizer interacts with the drug substance, while the hydrophilic chain extends into the aqueous phase. In electrostatic stabilization, repulsive forces between the charged nanocrystal particles are formed, which prevents particle aggregation.

Different strategies have been used to improve the solubility and oral bioavailability of poorly water-soluble drugs. These strategies include prodrug formation [14], cyclodextrins [15], cocrystals [16], co-amorphous solid dispersions [17], and eutectic mixtures [18]. Among the nanostructured drug delivery systems, drug nanocrystals are a prominent approach for enhancing the bioavailability of poorly water-soluble drugs. This carrier-free system consists of sub-micron drug particles (1–1000 nm) stabilized in a suspension, which can be suspended in either aqueous or non-aqueous media [19] (Figure 2). Compared with other nanostructured drug delivery systems, drug nanocrystals offer advantages such as high drug loading, improved long-term physical stability, ease of industrial scale-up, and the optional use of organic solvents [20].

Drug nanocrystals can be prepared using top-down, bottom-up, or combined methods. In the top-down approach, high input energy reduces the drug particle size to the nanoscale. Wet bead milling (WBM) and high-pressure homogenization (HPH) are the most effective techniques for obtaining drug nanocrystals via the top-down approach. In WBM, the micronized drug and a stabilizer solution are added to a milling container filled with wear-resistant beads, such as yttria-stabilized zirconia or cross-linked polystyrene. A high-speed rotor then reduces the drug particle size through shear, friction, and impact forces between the drug particles, the grinding medium, and the walls of the milling container [21]. In HPH, drug nanocrystals are produced by shear, turbulence, and cavitation forces as the coarse suspension (containing the drug, stabilizer, and vehicle) is forced through a narrow gap under pressure [22].

The bottom-up approach involves forming drug nanocrystals through nucleation in a supersaturated solution [23]. Precipitation or antisolvent precipitation is a typical bottom-up method used to obtain nanocrystals. A drug solution is prepared using an appropriate solvent (organic phase), while the stabilizer is dissolved in the antisolvent (aqueous phase) [19]. Adding the antisolvent to the solvent phase decreases the drug solubility, which creates supersaturation followed by nucleation and crystal growth [24,25]. A sonication step can be included to improve the mixing efficiency and facilitate drug nucleation [25].

All these techniques require the use of stabilizers to prevent physical instabilities, such as aggregation, sedimentation, and Ostwald ripening, which result from the increased Gibbs free energy when reducing the drug particle size to the nanoscale [26]. Using agents like non-ionic/polymeric or ionic stabilizers, or a combination of these, can effectively address these issues and maintain a nanocrystal particle size during storage (Figure 2) [26].

Due to its advantages, drug nanocrystals have been utilized in the development of innovative pharmaceutical products. The first marketed pharmaceutical product containing drug nanocrystals was Rapamune^®^ (sirolimus), which was launched in 2000 by Wyeth Pharmaceuticals (Madison, NJ, USA) [27]. The tablets with sirolimus nanocrystals showed a 21% increase in bioavailability compared with the marketed oral suspension. Emend^®^, which was launched in 2003 by Merck (Winehouse Station, NJ, USA), contains 80 mg or 125 mg of aprepitant nanocrystals, which represents a 25–30% dose reduction from conventional products. Tricor^®^ (Abbott, Wiesbaden-Delkenheim, Germany; 2004) and Megace ES^®^ (Par Pharmaceuticals, Spring Valley, NY, USA; 2005) eliminated the fed/fasted variation by presenting fenofibrate and megestrol acetate as nanocrystals, respectively [28].

The improvement of oral absorption and bioavailability through drug nanocrystals has been extensively documented. This review discusses the potential mechanisms involved, with a focus on nanocrystal properties and pharmacokinetic (PK) performance. Additionally, this review addresses the challenges associated with the oral administration of nanocrystals by highlighting opportunities for enhancing the bioavailability and efficacy of poorly water-soluble drugs.

Table 1 presents the recent findings from PK studies that involved drug nanocrystals. Most of these studies were conducted in rats due to their availability, relatively low cost, and ease of handling. Rabbits and dogs are also used as animal models for studying drug nanocrystal absorption, and PK results can be cautiously extrapolated to humans by taking into account species-specific differences. For instance, a drug that is well absorbed orally in humans often shows similar absorption in dogs, but the reverse may not always be true [29]. Humans and dogs have significant differences in their gastrointestinal tracts, including variations in the length, volume, composition, pH, and transit time [30,31,32].

## 2. Influential Factors for Drug Nanocrystals in Oral Absorption

**Figure 3:**
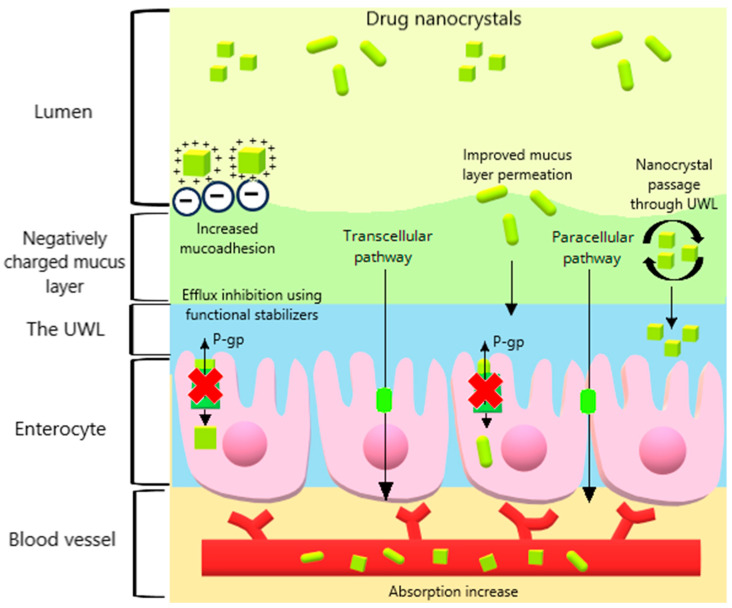
Schematic illustration of the opportunities provided by orally administered drug nanocrystals for enhancing intestinal absorption through various pathways. After oral administration, drug nanocrystals can improve the mucoadhesion, increase mucus permeation, and pass through the unstirred water layer (UWL) via the particle drift effect. Additionally, the use of functional stabilizers can inhibit the P-glycoprotein (P-gp) efflux and promote paracellular delivery. Subsequently, the high concentration gradient between the intestine and blood vessels enhances nanocrystal absorption.

Several factors influence the oral absorption of drug nanocrystals, including the particle size, dissolution rate, crystalline state, type and concentration of stabilizers, surface charge, and morphology. These factors contribute to enhancing the oral absorption through various mechanisms, such as improved mucoadhesion, minimized variability between fed and fasted states, enhanced permeation through the mucus layer, and increased intestinal transport (Figure 3).

### 2.1. Particle Size and Morphology

Table 1 presents drug nanocrystal particle sizes ranging from 46 to 540 nm. These preparations, which were obtained through top-down methods, varied from 151 to 522 nm, while bottom-up and combined-method strategies ranged from 46 to 540 nm and 84 to 420 nm, respectively (Table 1). This broad range of particle sizes can be attributed to the properties of the drug, the concentration of the drug and stabilizer, and the type of stabilizer used.

Additionally, each preparation method has specific variables that may influence the nanocrystal particle size. For WBM, these variables include the amount and size of the beads and the milling speed/time. For HPH, they include the homogenization pressure, number of cycles, and flow rate. For the precipitation technique, mixing efficiency, volume ratio, and flow rate of antisolvent/solvent are critical factors [52,53,54]. Understanding the critical process parameters, as well as the critical material attributes, is essential for the development of this preparation under a quality by design (QbD) approach. Statistical tools help to identify the interactions, critical factors, and significance of each variable on the nanocrystal particle size. In Table 1, 42% of the studies utilized these tools to optimize the nanocrystal size by employing methods such as the response surface methodology and fractional factorial design [33,40,42,44,47,48,49,50]. This provides a scientific rationale for understanding the particle size reduction as a function of independent variables.

The impact of the particle size in oral absorption was evaluated for cyclosporin A, which is an immunosuppressant, prepared as nanocrystals (280 nm, 522 nm, and 2967 nm) [45]. In the PK study, the reduction in particle size correlated with an increased C_max_ and AUC_0–48h_: 280 nm > 522 nm > 2967 nm. The AUC_0–48h_ value for the 280 nm cyclosporin A nanocrystal was 57.25 ± 0.92 h·µg/mL, which was 1.1- and 1.5-fold higher than that of the 522 nm and 2967 nm preparations, respectively. Additionally, the larger formulation (2967 nm) exhibited a 1.4-fold increase in T_max_, which indicates a potential agglomeration process that delayed the time to reach C_max_. Interestingly, even though the cyclosporin A nanocrystal at 280 nm showed an improvement in PK compared with the commercial product, the enhancement was modest. A further reduction in particle size below 280 nm could potentially yield more substantial PK improvements for cyclosporin A nanocrystals.

The impact of the particle size on oral absorption was also investigated using isoliquiritigenin as nanocrystals, which is an experimental flavonoid compound that has been reported to have anti-inflammatory and antitumoral activities [46]. Three formulations were prepared with particle sizes of 555.7 nm, 271.0 nm, and 46.2 nm, and their PK was evaluated in rats (Table 1). The preparations showed improvements in C_max_, T_max_, and AUC compared with coarse isoliquiritigenin. Surprisingly, the 271.0 nm nanocrystal exhibited the most significant enhancement in PK parameters, where it even surpassed the 46.2 nm nanocrystal (Table 1). This finding was consistent with the in vitro release from a dialysis membrane at 37 °C, where the preparations released 71.8%, 87.6%, and 80.9% within 48 h for the 555.7 nm, 271.0 nm, and 46.2 nm nanocrystals, respectively.

Notably, a smaller nanocrystal does not always translate to greater drug absorption. Similar scenarios were observed with coenzyme Q10, which is also known as ubiquinone and is a dietary supplement that can be used in the treatment of vascular disfunctions and other diseases [55]. The nanocrystal preparations that ranged from 700 nm to 120 nm demonstrated nearly identical bioavailability enhancement (~5-fold increase compared with a coarse suspension) until reaching a particle size of 80 nm, which resulted in a 7-fold increase in oral bioavailability [56]. This contrasts with dissolution tests in Tween 20 aqueous solution, where 80 nm and 120 nm nanocrystals showed only 65.4% and 13.2% release after 24 h, respectively. Nanocrystals sized at 400 nm and 700 nm, as well as pure coenzyme Q10, exhibited less than 5% release in the same timeframe.

These findings suggest that in vivo dissolution rates for nanocrystals may be rapid enough that they can overshadow differences in oral bioavailability between formulations with varying particle sizes. The high aqueous dissolution of drug nanocrystals can be enhanced by endogenous surfactant secretions, such as bile salts and phospholipids in the intestinal environment, as previously mentioned. Thus, achieving an optimal particle size is critical for each preparation to improve the oral bioavailability, which could pose a challenge during in vivo experiments. One approach to addressing this challenge is the use of simulation modeling to predict oral absorption based on specific particle sizes [57].

The particle size reduction of drug nanocrystals can also enhance mucus layer interactions, which can facilitate intestinal absorption. In the mucus layer, drug nanocrystals are well-suited to pass through this barrier, as they fall within the mucus pore size range of 50–1800 nm [10]. The passage through the mucus layer involves adsorption and penetration mechanisms. During adsorption, interactions between nanocrystals and mucus may occur, which can prolong the drug retention time, increase the concentration gradient, and has the potential to promote drug diffusion [10]. The penetration of drug nanocrystals in the mucus layer is estimated by the Stokes–Einstein equation (Equation (1)), where the radius of the diffusing particles (r) is inversely proportional to the diffusion coefficient (D) [58]:

(1) $$D=\frac{kT}{6\eta \pi rh}$$

where k is the Boltzmann constant, T is the absolute temperature, η is the viscosity of the mucus layer, and h is the thickness of the mucus layer. The morphology of nanocrystals also plays a significant role in mucus permeation and absorption enhancement. Lovastatin nanocrystals, a lipid-lowering drug, were obtained with distinct morphologies and have been reported to exhibit different bioavailabilities in rats (Table 1) [35]. Similarly, in a mucus permeation study using rat intestinal mucosa, rod-shaped lovastatin nanocrystals demonstrated greater penetration in the mucus layer compared with flaky and spherical nanocrystals. The rod-shaped nanocrystals were also more easily internalized by epithelial cells during an in situ intestinal absorption test. The study related this result to the fact that spherical lovastatin nanocrystals are more likely to be trapped in the mucus layer networks, which reduces their apparent permeability coefficient (P_app_) compared with rod-shaped nanocrystals. The larger contact area of rod-like nanocrystals with the intestinal membrane, combined with their ease of rotation through mucus networks, may facilitate the permeation and internalization of rod-like nanocrystals compared with other morphologies [35,59].

### 2.2. Dissolution Rate

The unique properties exhibited by drug nanocrystals are demonstrated by mathematical models. According to the Noyes–Whitney equation (Equation (2)), the large surface area-to-volume ratio and the short diffusion distance result in an increase in the dissolution rate [60]:

(2) $$\frac{dc}{dt}=\frac{D\times A\times (Cs-Cx)}{h}$$

where dc/dt is the dissolution rate (drug concentration shift by time), D is the diffusion coefficient, A is the surface area-to-volume ratio, Cs is the saturation concentration, Cx is the bulk concentration, and h is the diffusional distance.

The drug in the nanometer range also affects the saturation solubility since it increases the particle curvature, which enhances the dissolution pressure and shifts the equilibrium toward dissolution [61]. This phenomenon is parallel with the Kelvin equation (Equation (3)), where the particle radius is inversely proportional to the dissolution pressure [23,61]:

(3) $$\ln\frac{Pr}{P}=\frac{2\gamma Mr}{rRT\rho }$$

where Pr is the dissolution pressure of a particle with the radius r, P∞ is the dissolution pressure of a large particle, γ is the surface tension, Mr is the molecular mass, r is the particle radius, R is the gas constant, T is the absolute temperature, and ρ is the particle density. Consequently, more particles can interact with aqueous biological fluids, and the concentration gradient between the intestinal membrane and blood vessels is improved. Then, drug absorption and oral bioavailability can be enhanced.

Approximately 81% of the studies (Table 1) conducted in vitro dissolution tests. Among these studies, 88% reported an enhanced dissolution rate of drug nanocrystals compared with the control. As elucidated by the Noyes–Whitney equation, the improvement in dissolution rate is attributable to the reduction in the particle size to the nanoscale. This size reduction increases the surface area-to-volume ratio and decreases the diffusion layer thickness surrounding the drug particles, thereby facilitating nanocrystal dissolution. The rapid dissolution enabled by drug nanocrystals is essential for improving the oral bioavailability of poorly water-soluble drugs.

For example, in vitro dissolution studies of gliclazide as nanocrystals, which isan antidiabetic drug, in a phosphate buffer (pH 7.4) at 37 °C showed a 2.3-fold higher release compared with the pure drug [44]. This increase is attributed to the larger surface area-to-volume ratio achieved by reducing the drug particle size to the nanoscale. Significant improvements in C_max_ and AUC_0–t_ for gliclazide nanocrystals were presented in the PK studies (Table 1), while the time to reach C_max_ (T_max_) was 3-fold shorter than that of the pure drug. In rats subjected to a glucose overload (2 g/kg orally), drug nanocrystals showed a remarkable reduction in glucose blood levels compared with the pure drug and commercial product. These results show the importance of dissolution rate enhancement by drug nanocrystals in achieving rapid drug absorption and potentially improving the efficacy.

In addition to enhancing the oral bioavailability, the increased dissolution rate and saturation solubility provided by drug nanocrystals can significantly benefit poorly water-soluble drugs that are affected by variations between fed and fasted states. As previously mentioned, absorption variability in these states can lead to sub-optimal drug levels or toxicity, especially for drugs with a low therapeutic index. Drug nanocrystals are known for their potential to eliminate fed/fasted state variations due to the dissolution rate in both conditions being fast enough [62]. In the PK studies, similar AUC values were observed in both the fed and fasted groups following the oral administration of nanocrystals [33,63,64,65].

Entrectinib is an anticancer drug that exhibits decreased solubility under fasted conditions [66]. Entrectinib is a weak base (pKa 2.5), and it presents higher solubility in gastric fluid in the absence of food but tends to precipitate upon reaching the intestine [50]. To address this issue, an entrectinib nanocrystal (83 nm) was developed, and the food effect was evaluated in rats [50]. Entrectinib nanocrystals exhibited a dissolution rate of approximately 98% after 4 h in both fasted- and fed-state simulated intestinal fluids (FaSSIF and FeSSIF), in contrast to the dissolution rates of 74% and 61% for pure entrectinib, respectively. This observation is consistent with the PK results, which demonstrated nearly equivalent AUC_0–24h_ values for the nanocrystals in both states (13,949.9 ± 329.3 ng·h/min in the fed group vs. 11,702.8 ± 330.6 ng·h/min). These results suggest the elimination of the fed/fasted variability. Furthermore, the C_max_ and AUC_0–24h_ of the nanocrystals increased by 4-fold and 3-fold, respectively, compared with pure entrectinib (Table 1).

Nevertheless, the fed/fasted state variation may not be eliminated by the nanocrystal approach. For example, ritonavir nanocrystals (541.8 nm), an antiretroviral drug, were developed, and the food effect was evaluated in rats [34]. In a dissolution test at 37 °C using 0.06 M polyoxyethylene 10 lauryl ether media, the coarse drug, commercial product, physical mixture, and nanocrystals all achieved 100% release after 2 h. The nanocrystals improved the AUC_0–8h_ in the fed group when compared with the commercial product (Table 1), coarse drug, and physical mixture. In the fasted group, the commercial product presented a slightly higher (1.3-fold) AUC_0–8h_ than the nanocrystal.

When comparing the nanocrystal group performance in the fed and fasted states, absorption in the fed group was almost 10 times higher than in the fasted group (AUC_0–8h_ of 442.3 ± 244.4 min·µg/mL vs. 47.7 ± 15.9 min·µg/mL, respectively). This outcome indicates that the improvement in oral bioavailability provided by nanocrystals was not sufficient to eliminate the food effect. This suggests that the drug’s intrinsic properties can override the benefits provided by nanocrystals. In the fasted state, the gastric pH of healthy humans typically ranges from 1 to 3 [67]. However, when the pH shifts to 6–8 in the duodenum due to bicarbonate buffer secretion, pH-dependent drugs can precipitate in the small intestine [5]. Given that ritonavir is a weakly basic drug, supersaturation in the stomach and subsequent precipitation in the intestine is anticipated [34]. Additionally, magnetic resonance imaging data show that in the fasted state, the gastric fluid content is less than 50 mL, and the small intestine is mostly empty [5]. This limited fluid volume restricts drug solubilization in the gastrointestinal tract in the absence of food.

When correlating the PK performance of ritonavir nanocrystals with entrectinib nanocrystals—both of which are weakly basic drugs—entrectinib nanocrystals were able to eliminate the variability between the fasting and fed states, as previously mentioned, unlike ritonavir nanocrystals. This suggests that entrectinib may be less dependent on endogenous surfactant secretion for solubilization in the fed state compared with ritonavir. The difference in particle size between the two preparations (ritonavir nanocrystals at 541.8 nm and entrectinib nanocrystals at 83 nm) also indicates that reducing the particle size of ritonavir nanocrystals might be necessary to achieve the dissolution rate required to eliminate the variability between the fasting and fed states. Additionally, since the elimination of food effects by nanocrystals is attributed to their rapid dissolution regardless of food presence, the absorption of these formulations may be limited by permeability [68]. Considering that ritonavir is a BCS class IV drug and entrectinib is a BCS class II drug, the differences observed in the fed/fasted state studies may be related to ritonavir’s poor permeability, which may not have been fully addressed by the nanocrystal approach [69,70,71].

Notably, significant differences were observed in the PK performance of the commercial ritonavir product (Norvir^®^, Abbott, Chicago, IL, USA) in male Wistar rats compared with humans. Similar phenomena may occur with ritonavir nanocrystals. Following a 10 mg/kg oral administration in rats, the commercial product exhibited a positive food effect (AUC_0–8h_ of 63.2 ± 25.1 min·µg/mL in the fasted state and 221.9 ± 98.7 min·µg/mL in the fed state). Conversely, after an oral administration of 100 mg of ritonavir in healthy humans, a negative food effect was observed (AUC of 4.6 ± 2.0 min·µg/mL in the fasted state and 3.5 ± 1.6 min·µg/mL after food intake) [72,73]. This negative food effect in humans was supported by different study [74]. In addition to differences in the administered doses, significant anatomical and physiological differences between humans and rats may impact drug absorption. The human intestinal tract is approximately 5.5 times longer than that of rats, resulting in a surface area that is 200 times larger [75]. Nevertheless, the considerable difference in body size between humans and rats renders this intestinal surface area not directly comparable [75]. Furthermore, theoretical data suggest that while the rat model can be useful for predicting oral drug absorption in humans, it is not reliable for predicting bioavailability [76]. Therefore, it is necessary to conduct studies that involve drug nanocrystal absorption in the target species to clarify improvements in oral bioavailability, especially during fed/fasted state studies.

These species-specific differences in food effect evaluation may also be observed for megestrol acetate nanocrystals. In male beagle dogs, after the oral administration of 10 mg/kg in the fed state, megestrol acetate nanocrystals exhibited an AUC_0–t_ that was 1.6 times higher than in the fasted state (*n* = 3) [77]. Compared with the commercial product, the nanocrystal formulation showed a greater enhancement in AUC_0–t_ in the fasted state (2-fold increase), while the bioavailability was similar in the fed state. In a randomized, open-label, crossover study conducted in healthy male humans, a single 625 mg/5 mL dose of megestrol acetate nanocrystals in the fasted state demonstrated bioequivalence to the commercial product (https://clinicaltrials.gov/study/NCT06147908, accessed on 17 August 2024) (*n* = 52) [78]. Beagle dogs are commonly used in preclinical studies to assess the oral absorption of drug delivery systems intended for human use due to their similar gastrointestinal tract dimensions and comparable fasted-state gastric motility [30,79]. However, contrary to expectations, the fasted state in dogs often limits drug absorption since dogs typically do not voluntarily drink water after receiving oral medication, which can restrict the drug dissolution in the limited residual gastrointestinal water [80]. Despite this, the gastric pH in fasted dogs is reported to range from about 2 to 6.5 due to lower basal peak acid secretion, which may allow weakly acidic drugs to dissolve more easily, and thereby enhance the drug absorption [31]. Therefore, it is highly recommended the use of compounds, such as pentagastrin, in dog models to decrease the gastric pH and better mimic human conditions during PK studies to minimize these species-specific differences [79].

Regarding the food effect, another important case is cinacalcet as nanocrystals, which is a drug used in the treatment of hyperparathyroidism [33]. The cumulative dissolution of cinacalcet nanocrystals exceeded 95% in water and buffer solutions at pH 1.2, pH 4.5, and pH 6.8. In contrast, the pure drug exhibited less than 20% dissolution in these four different media. The commercial product (Sensipar^®^ tablets, Amgen Inc., Thousand Oaks, CA, USA) demonstrated a dissolution profile similar to that of the nanocrystals in pH 1.2 and pH 4.5 buffers. The fed/fasted variation was assessed in rats, and the similar AUC_0–t_ values in both states indicate that the cinacalcet nanocrystals eliminated the food effect (Table 1). Compared with the commercial product, the nanocrystals showed improvements in C_max_ and AUC_0–t_ in the fasted group. In the fed group, the cinacalcet nanocrystals presented similar C_max_ and AUC_0–t_ values to the commercial product, which could be correlated with the dissolution rate. Lipophilic drugs administered with high-fat meals tend to have an improved solubility and absorption in the small intestine due to increased bile salt secretion in response to food intake [81]. In the fed state, the rate of gastric emptying is reduced, which enables the accumulation of substantial volumes of food and water within the gastric lumen. This accumulation acts as a dissolution medium for oral dosage forms [5]. Furthermore, pharmaceutical excipients present in solid oral dosage forms, such as disintegrants, high concentrations of surfactants, sugar-containing excipients, and natural polymers, can enhance the aqueous solubility of drugs [2]. This can explain the similarity in C_max_ and AUC_0–t_ values between the cinacalcet nanocrystal and the commercial product in the fed group.

Additionally, drug nanocrystals offer a significant advantage by improving the dissolution rate and saturation solubility, then creating a concentration gradient across the intestinal membrane that promotes transcellular diffusion via passive transport [10] (Figure 3). Nanocrystals were shown to increase the drug transport across various cell models. Ginkgolide B, which is an experimental compound derived from the *Ginkgo biloba* tree used in Parkinson’s disease, was obtained as nanocrystals (85 nm) [36]. The preparation showed a 92.17% release at pH 7.4 in phosphate buffer saline (PBS) after 30 min compared with 16% for pure ginkgolide B. [36]. The transport of these nanocrystals through Mardin–Darby canine kidney (MDCK) cells presented a P_app_ that was 2.7-fold higher than that of pure ginkgolide B without compromising the integrity of the MDCK monolayer. The PK studies in rats revealed that the nanocrystals significantly increased the plasma C_max_ and AUC_0–t_ compared with the pure compound (Table 1). In neuronal tissue following the oral administration, this increase was 3-fold for C_max_ and 2.5-fold for AUC_0–t_, which indicates that nanocrystals could be a promising strategy for brain drug delivery after oral administration. This result is attributed to the small particle size of the nanocrystals, which allows the drug to cross the blood–brain barrier (BBB). The use of HPMC E5 as a stabilizer may also play a role, as its osmotic properties can enhance drug concentration in brain tissue [82,83]. However, orally administered drug nanocrystals will likely dissolve in the gastrointestinal tract before crossing the BBB as intact particles, which makes the ideal particle size for passing through this barrier negligible.

Some reports suggest that the enhanced absorption of drug nanocrystals is primarily due to increased permeation rather than an increased dissolution rate. Megestrol acetate nanocrystals, an appetite stimulant, were evaluated in a dissolution–permeation study using a setup with donor (pH 6.5) and acceptor (pH 7.4) chambers at 37 °C that were separated by a PVDF membrane [37]. A drug solution in methanol was also tested to determine the impact of dissolution on permeability. The results showed that the P_app_ increased with nanocrystal particle size reduction (158 nm = 200 nm > 373 nm > 1950 nm). The driving force of membrane permeation is the reduction of drug concentration from the donor to acceptor chambers over time. If the permeation depends on dissolution, the megestrol acetate solution would have a higher permeation rate than the drug nanocrystals (which are suspensions). However, the P_app_ of nanocrystals (158 nm) was found to be 2-fold higher than that of the drug solution.

In PK studies in rats, the 158 nm megestrol acetate nanocrystals showed a 3.6, 1.4, and 1.8-fold improvement in AUC_0–2h_ compared with the microsuspension (1950 nm), commercial product (200 nm), and coarse nanocrystal (373 nm), respectively (Table 1). These results may indicate that permeation, not dissolution, is the key factor for the improved oral absorption of megestrol acetate nanocrystals. The authors related this result to the reduction in the effective membrane thickness in the UWL. Colloidal drug particles, such as nanocrystals, are reported to improve passive permeation through the UWL using a mechanism termed the particle drifting effect [84]. The mucus layer in the UWL can be divided into firmly and loosely adhered regions, and the particle-drifting effect occurs when drug particles smaller than 2 µm drift in both regions [11,85] (Figure 3). Once within the UWL, drug nanocrystals have the potential to form a reservoir to be dissolved in the UWL, which allows drug molecules to diffuse to the epithelial cell membrane [84]. When the distance between the drug particles in the UWL and the epithelial cell membrane is smaller than the distance between the bulk fluid and the UWL interface, the effective thickness of the UWL (i.e., diffusional resistance) is reduced [11]. Since the UWL barrier dominates the permeation resistance of BCS class II drugs, nanocrystals can provide an additional mechanism for improving intestinal absorption through the particle-drifting effect.

According to the Biopharmaceutics Classification System (BCS), the extent of drug absorption is primarily determined by the transport of the drug from the intestine into the body, mainly across the epithelial cell membrane surface of the gastrointestinal tract, without accounting for chemical or enzymatic degradation [86,87]. This establishes a correlation between the extent of the drug absorption and the intestinal membrane permeability. However, since megestrol acetate is a BCS class II drug with poor aqueous solubility but high permeability, the primary limiting factor for its oral absorption is the dissolution rate, not its permeability [88]. This concept is more relevant to BCS class IV drugs, which have both poor solubility and poor permeability, where improving the dissolution alone, without also enhancing permeability, may have little impact on the oral absorption [89]. For some BCS class IV drugs, simply enhancing the dissolution through nanocrystals may not effectively improve the oral bioavailability [71]. PK studies that compared nanocrystals to a drug solution administered orally could provide further insight into the role of permeability in the absorption of megestrol acetate nanocrystals.

In cell-based permeation studies, the dissolution reflects the driving force for drug nanocrystal absorption. Nintedanib, which is used to treat pulmonary diseases, was prepared as nanocrystals (325.3 ± 1.0 nm) [39]. The preparation showed a 1.3-fold lower transport through human colon adenocarcinoma (Caco-2) monolayers compared with the drug solution in Tween 80. In contrast, the in situ single-pass intestinal perfusion in rats showed that nanocrystals improved absorption in all intestinal segments compared with the solution. The PK studies also revealed that nintedanib nanocrystals increased the AUC_0–t_ by 2.5-fold compared with the drug solution (Table 1). This suggests that for these nanocrystals, dissolution may be the primary factor for enhanced absorption rather than permeability. Drug nanocrystals released 73.7% at pH 6.8 within 10 min compared with 6.1% for the pure drug. The lower transport of nanocrystals through cells compared with the drug solution is attributed to reservoir formation in the mucus layer, which reduces the effective diffusion thickness of the drug molecules. An alternative explanation could be that nintedanib nanocrystals may need to dissolve before being transported by Caco-2 monolayers, which is the possible reason why the drug solution presented superior transport over the drug nanocrystals.

### 2.3. Crystalline State

The molecular arrangement of drugs in crystalline or amorphous structures significantly influences their aqueous solubility. While the term “nanocrystals” typically suggests a nanoparticulate system in a crystalline state, drug nanocrystals involve a particle size reduction, whether in a crystalline or amorphous form. Generally, crystalline forms exhibit regular atomic patterns and stable molecular conformations that reduce the Gibbs free energy, whereas amorphous compounds have randomly arranged molecules [90].

Combining a particle size reduction with an amorphous state showed notable results in enhancing the solubility and dissolution rate of poorly water-soluble drugs, which consequently increased the drug absorption and oral bioavailability. Cilnidipine, which is an antihypertensive drug, was prepared as a nanocrystal, and X-ray diffraction analysis confirmed its amorphous state [40]. The nanocrystal achieved 85% release within the first 5 min compared with the pure drug. In saturation solubility testing, the nanocrystal showed a 6.9-fold increase in a phosphate buffer (pH 6.8) compared with the pure drug. These results align with the PK study, where the cilnidipine nanocrystals exhibited a significantly increased C_max_ and AUC_0–t_ compared with both the pure drug and the commercial product (Table 1). Similarly, venetoclax nanocrystals, which are used in the treatment of lymphocytic leukemia, were found to be partially amorphous due to the high pressure used during their preparation [38]. Venetoclax nanocrystals demonstrated a remarkable 20-fold increase in saturation solubility at pH 6.8 (sodium phosphate buffer) compared with the free base drug. Dissolution testing revealed the complete release of drug nanocrystals (100%) within 2 h, in contrast to the < 43.5% for conventional venetoclax. The PK studies in rats showed a 2-fold increase in the relative oral bioavailability and a 1.7-fold increase in C_max_ compared with the commercial product (Table 1).

The superior saturation solubility and dissolution rate of amorphous nanocrystals compared with their crystalline counterparts stem from weaker bonding forces between molecules [90]. It requires less energy to disrupt the drug’s internal structure and release isolated molecules into the supersaturated state [91]. However, a significant challenge in amorphous nanocrystals is maintaining the supersaturated state before nucleation and crystal growth. An effective stabilizer system is crucial to address this challenge and ensure that the drug remains at adequate concentrations throughout its gastrointestinal transit.

### 2.4. Stabilizing Agents

As previously mentioned, the increase in Gibbs free energy caused by particle size reduction will tend to cause drug nanocrystals to agglomerate or undergo Ostwald ripening to decrease this energetic state [92]. Therefore, the use of stabilizers is crucial to prolong the physical stability of drug nanocrystals during storage. Most reports in Table 1 used non-ionic/polymeric stabilizers, followed by combinations of non-ionic/polymeric + ionic stabilizers. Non-ionic/polymeric agents stabilize drug nanocrystal by forming a steric barrier between the particles, while ionic stabilizers provide electrostatic stabilization by creating repulsive forces between the charged particles [93]. The frequent use of non-ionic/polymeric stabilizers in Table 1 can be attributed to advantages such as their effectiveness in both aqueous or non-aqueous media and their insensitivity to pH changes in the gastrointestinal tract [52]. The combination of non-ionic/polymeric + ionic stabilizers has gained prominence for stabilizing drug nanocrystals through both steric and electrostatic mechanisms, which can minimize the particle size variability during storage.

In addition to this primary role, the stabilizers can contribute to the oral bioavailability of drug nanocrystals. For instance, the effect of stabilizing agents on the dissolution rate, saturation solubility, and oral absorption in rats of quercetin nanocrystals, a supplementary flavonoid with anti-inflammatory and antioxidant activities, was evaluated [51]. The stabilizers tested were HPMC E15, poloxamer 407, poloxamer 188, TPGS, and glycyrrhizin acid (GL). Quercetin nanocrystals were prepared using equal amounts of the drug and each stabilizer, which resulted in similar particle sizes (~200 nm) to eliminate the size as a variable in drug nanocrystal performance. All nanocrystals improved the saturation solubility of quercetin in water, with the most significant enhancements for nanocrystals stabilized with P407 and TPGS. These preparations showed 16- and 14-fold increases, respectively, compared with pure quercetin.

In dissolution tests, the release order of the nanocrystals was P407 > TPGS > GL > P188 > HPMC E15 > pure quercetin. Interestingly, the increase in AUC_0–t_ compared with pure quercetin was as follows: HPMC E15 = P188 > GL > P407 = TPGS (Table 1). Similar to the AUC_0–t_ results, all nanocrystals increased C_max_ compared with pure quercetin, with the most pronounced enhancement observed in nanocrystals stabilized with P407 (9-fold). These stabilizers prolonged T_max_, except P407 and TPGS, which provided T_max_ values that were 6- and 3.9-fold faster than pure quercetin. This finding aligns with the greatest increase in saturation solubility and dissolution rate for these preparations. Although the benefits of drug nanocrystals are typically attributed to improved dissolution rates and aqueous solubility, this study demonstrated that stabilizing agents significantly impact the PK performance of orally administered nanocrystals. Further investigation is required to fully understand the functional roles of each stabilizer and determine which effects will be the primary driving forces following the oral administration of drug nanocrystals.

The stabilizing agent can also affect drug nanocrystal transport in intestinal cells. Drug transport across the intestinal membrane involves both transcellular and paracellular diffusion. Paracellular diffusion refers to drug absorption through tight junctions with pore sizes not exceeding 20 nm, which significantly limits drug nanocrystal transport via this pathway [10]. The paracellular delivery for nanocrystals can be enhanced by using stabilizers that open tight junctions, such as sodium dodecyl sulfate (SDS), Kolliphor^®^ HS15, sodium deoxycholate, and chitosan [41,94,95,96] (Figure 3). Additionally, some stabilizing agents are reported to avoid drug efflux by the P-gp pump. TPGS, which is a non-ionic stabilizer derived from vitamin E, is known for modulating the P-gp efflux transporter by blocking sites in the P-gp pump and preventing drug efflux (Figure 3) [97,98]. Similar to TPGS, Soluplus^®^, Tween 80, Cremophor^®^ EL, and poloxamers are commonly used as stabilizers in drug nanocrystal preparations and are P-gp inhibitors [43,47,48,99,100]. Using P-gp inhibitors in nanocrystals is particularly attractive for cancer chemotherapy, as P-gp overexpression causes multidrug resistance [101,102]. Additionally, a high drug concentration in the intestinal lumen can saturate the P-gp pump, which reduces the efflux and promotes absorption, which is a condition that drug nanocrystals can easily achieve [13]. For example, nilotinib, which is a BCS class IV drug used in leukemia treatment and a P-gp substrate, showed significantly improved PK parameters when prepared as a nanocrystal with Soluplus^®^ as the stabilizer (Table 1) [47]. This effect is likely attributable to the increased passive diffusion, the particle drifting effect through the UWL, and P-gp inhibition by Soluplus^®^, despite further studies being required to address this mechanism for nilotinib nanocrystals.

### 2.5. Surface Charge

The electrostatic stabilizers can provide a high positive (cationic) or negative (anionic) surface charge, which can be utilized to improve the oral absorption of drug nanocrystals, in addition to their stabilization role. For instance, electrostatic attraction forces between the drug surface and the mucus layer can further prolong the drug retention time, which enhances mucoadhesion [103] (Figure 3). The glycosylated amino acids by glycan linkages present in the mucus layer contribute to the negative charge density present in mucins [104]. This property can be exploited in drug nanocrystal development by using cationic stabilizers, which provide positive charges that increase the electrostatic attraction forces between the opposite charges of nanocrystals and the mucosa (Figure 3).

Aprepitant nanocrystals, which are used as an antiemetic, were prepared by wet ball milling using hydroxypropyl chitosan as a stabilizer [41]. This chitosan derivative has a carboxyl group with Ca^2+^ binding ability, which can remove divalent ions from the extracellular matrix and increase permeability [41,105]. According to PK results, the aprepitant nanocrystal presented 3.4-fold and 1.5-fold increases in AUC_0–t_ compared with the pure drug and commercial product, respectively. The cationic stabilizer provided a high positive charge to the aprepitant nanocrystal (+63.5 ± 0.3 mV), which could enhance the nanocrystal mucoadhesion and enable continuous drug release at the mucosal surface, and thereby promote bioavailability.

However, the mucoadhesion strength requires further investigation, as some reports indicate that a very positive surface charge can strongly interact with mucin. This can cause drug nanocrystals to have difficulty penetrating the mucus layer, thereby hindering drug absorption [103,106,107]. For instance, bufadienolides are experimental compounds derived from traditional Chinese medicine and are explored in cancer treatment [108]. Bufadienolide nanocrystals with different stabilizers were prepared, and their mucus permeation was evaluated using a transwell system that contained intestinal mucus from rats [108]. The cumulative penetration of nanocrystals was significantly higher than that of the conventional compound, especially for the pectin-stabilized formulation (209.2 ± 4.6 nm, ZP: −30.5 ± 1.7 mV). Despite this general increase, nanocrystals stabilized with chitosan quaternary ammonium salt (438.2 ± 1.5 nm, ZP: +30.0 ± 1.8 mV) presented the lowest P_app_. This result suggests that the high positive charge of this preparation promoted a strong interaction with mucus, thus compromising nanocrystal penetration. Additionally, the larger particle size of this cationic-stabilized nanocrystal could also contribute to its reduced penetration compared with negatively charged bufadienolide nanocrystals.

While mucoadhesion can enhance drug absorption, its effect may be negligible during oral delivery due to the rapid dissolution of drug nanocrystals. This challenge can be addressed by coating drug nanocrystals with polymer-based carriers and hydrogels to improve the local retention. Simvastatin nanocrystals, which are used in the treatment of dyslipidemia, were prepared and coated with thiolated xanthan gum to enhance mucoadhesion in the upper gastrointestinal tract [42]. In dissolution tests, the nanocrystals showed superior drug release compared with pure simvastatin in both pH 2.0 and pH 7.0 media. Drug nanocrystals presented a very high ZP value in the module (+20,000 mV), which was probably due to thiolated groups (R–S^−^) present in the xanthan gum coating that could increase disulfide bonds within the polymeric system of simvastatin nanocrystals [42]. In a mucoadhesion study that used 0.1% mucin, the mucoadhesive nanocrystals showed a decreased ZP value over time that reached +16,500 ± 869 mV after 6 h. The decrease in zeta potential during incubation with mucin was related to the interaction between the positive charges on the nanocrystal surface and the negatively charged mucin.

After oral administration in rats, the mucoadhesive simvastatin nanocrystals significantly increased T_max_, as well as AUC_0–∞_ by almost 22 times compared with the pure drug (Table 1). The possibly high mucoadhesion capacity led to a C_max_ that was 2-fold lower than pure simvastatin. This strong mucoadhesion also may have resulted in dose-dependent cytotoxicity during the MTT test, which was potentially related to the remarkable increase in MRT from 23 h to 96 h in the nanocrystal group. Therefore, it may be necessary to evaluate alternative coating systems to provide mucoadhesion that improves the drug retention time while ensuring patient safety.

## 3. Pharmacokinetics Particularities

Drug nanocrystals usually present significant improvements in C_max_, T_max_, and AUC, as shown in Table 1. However, the half-life (t_1/2_) can be prolonged with nanocrystals. A study on dolutegravir nanocrystals in rats showed that the nanocrystals exhibited increased C_max_ and AUC, along with a 1.4-fold higher t_1/2_ compared with the pure drug (Table 1) [48]. The extended t_1/2_ from 18.4 ± 1.2 h to 25.2 ± 3.3 h can be advantageous for dolutegravir, which is an antiretroviral drug, as prolonged systemic exposure can address challenges such as adherence failures, costly treatment, and frequent administration [109]. Although the increase in t_1/2_ in this study was slight, coating the preparation with polymers or hydrogels is a potential alternative to prolong nanocrystal concentration in the bloodstream and viral tissue reservoirs.

Similarly, mangiferin, which is an experimental polyphenol compound derived from the mango tree with antioxidant and anti-inflammatory activities, was prepared as nanocrystals that combined HPMC, poloxamer 407, and Tween 80 as stabilizing agents [49]. The PK study in rabbits demonstrated prolongations of 1.5-fold in t_1/2_ and 2-fold in T_max_, slight increases in C_max_ and AUC_0–t_, and a 1.4-fold enhancement in MRT. This indicates that the mangiferin nanocrystals exhibited prolonged systemic exposure, which was likely due to a coating effect from the stabilizer combination. The reduced intensity of the endothermic peak of mangiferin in the drug nanocrystals suggests either dissolution upon heating or coating by the stabilizers. This finding is corroborated by the in vitro dissolution study in pH 7.4 phosphate buffer, where the preparation achieved almost 100% release only after 8 h of testing.

However, a longer t_1/2_ can also raise concerns about potential toxicity, which is a significant consideration for nanostructured drug delivery systems. Nanocrystals can penetrate deeper into tissues than larger particles and remain in the organism for a longer duration [110]. The internalization of nanocrystals by cellular epithelium and endothelium plays a role in this aspect [111]. Nevertheless, this toxicity is typically limited to drug nanocrystals smaller than 100 nm, and the recognition of orally delivered nanocrystals by these cells is minimal since dissolution occurs quick enough before absorption [23].

Unexpected PK performance for drug nanocrystals can occur. Rebamipide nanocrystals, a quinolone drug used to treat gastritis, were prepared and incorporated into a bilayer tablet (Table 1) [43]. This bilayer tablet consisted of rebamipide nanocrystals for immediate release and a sustained-release layer of the conventional drug (total dose: 300 mg), with the aim to decrease dosing frequency. The dissolution of the bilayer tablet was conducted in two stages: using a pH 1.2 buffer for 2 h, followed by a pH 6.8 buffer for 24 h. Almost 60% of rebamipide nanocrystal was released in the first 2 h at pH 1.2, and it reached 100% after 24 h at pH 6.8. Surprisingly, no considerable differences in oral absorption between the bilayer tablet and the commercial product were observed in the PK study with beagle dogs, even though the bilayer tablet dose was three times that of the commercial product (300 mg vs. 100 mg, respectively). The authors attributed this result to rebamipide’s absorption site being in the upper gastrointestinal tract, which causes sustained release in the intestine inappropriate [43,112]. This challenge may be overcome by designing a gastroretentive drug delivery system that allows rebamipide nanocrystals to remain in the upper gastrointestinal tract for a longer duration [113].

Another possible reason for the lower bioavailability of the nanocrystals compared with the commercial product is the potential removal of nanocrystals from the bloodstream by macrophage phagocytosis [114]. Once inside macrophages, drug nanocrystals can take up to 20 days to release, which compromises absorption [115]. However, this effect is negligible for oral administration because the rapid dissolution of nanocrystals mitigates such uptake, whereas it is more pronounced in intravenous drug delivery [114].

## 4. Conclusions

The enhanced oral absorption and bioavailability provided by drug nanocrystals are primarily attributed to their reduction in particle size to the nanoscale. This enhancement can be further improved when the nanocrystals are in an amorphous state, although maintaining the physical stability of such preparations is a concern. Pharmacokinetic studies may not always differentiate between drug nanocrystals with different particle sizes, which suggests the existence of an optimal particle size range for significant bioavailability improvement for each nanocrystal preparation. The dissolution rate can be fast enough to eliminate the absorption variability between the fed and fasted states, although this effect can be influenced by the drug’s intrinsic properties, such as pKa. While nanocrystals can enhance the transcellular delivery, whether the primary driving force for their absorption is permeation or the dissolution rate needs further investigation. The use of functional stabilizing agents can improve nanocrystal absorption by enhancing the mucoadhesion, opening tight junctions, and inhibiting the P-gp efflux transporter. The increased surface area-to-volume ratio of nanocrystals can enhance their interaction with the mucus layer, especially when using a cationic stabilizer. However, overly strong interactions can hinder drug absorption or cause toxicity. Overall, drug nanocrystals have proven to be an important strategy for overcoming challenges associated with the oral administration of poorly water-soluble drugs while considering the formulation’s particularities and limitations.

## Figures and Tables

**Table 1:** Key pharmacokinetics of orally delivered drug nanocrystals in studies conducted from 2019 to 2024 that involved rats, rabbits, and beagle dogs.

Drug Nanocrystal			In Vivo Performance		
Composition	Preparation Method	Particle Size (nm)	Control	Main Observations	References
Cinacalcet Soluplus^®^	Antisolvent precipitation	244 ± 2	Commercial product	Increase by 2-fold in C_max_ and 1.5-fold in AUC_0–t_ in the fasted state; elimination of food effect	[33]
Ritonavir HPMC 3cps + SDS	HPH	541.8 ± 14.5	Stabilizer solution Coarse suspension Physical mixture Commercial product	Increase by 2-fold in C_max_ and AUC_0–8h_ compared with commercial product in the fed state	[34]
Lovastatin P188	Spherical: WBM Rod: antisolvent precipitation–HPH Flaky: cooling crystallization–HPH	405.8–417.3	Drug solution in ethanol and Cremophor^®^ EL	Rod-shaped increased AUC_0–24h_ by 1.8-fold and 1.4-fold compared with other morphologies	[35]
Ginkgolide B HPMC E5	Antisolvent precipitation	83.5 ± 1.8	Active compound suspended in HPMC	Increase by 13-fold in C_max_ and 5-fold in AUC_0–t_	[36]
Megestrol acetate HPMC + SDS	WBM	158.0	Microsuspension Commercial product Coarse nanocrystals	Increase by 2.7-fold in C_max_ and 3.6-fold in AUC_0–2h_ compared with microsuspension	[37]
Venetoclax PVA	Antisolvent precipitation–HPH	~340.0	Drug suspension	Increase by 1.8-fold in C_max_ and 2-fold in AUC_0–∞_	[38]
Nintedanib Na-CMC	Antisolvent precipitation	370.3 ± 19.3	Drug suspension in T80	Increase by 2.5-fold in AUC_0–t_	[39]
Cilnidipine P188 + T80	Antisolvent precipitation	280.1 ± 3.7	Drug suspended in Na-CMC Commercial product	Increase by 3-fold in C_max_ and 1.8-fold in AUC_0–t_ compared with the commercial product	[40]
Aprepitant HPCS	WBM	151.0 ± 14.5	Active compound suspended in Na-CMC Commercial product	Increase by 1.3-fold in C_max_ and 1.5-fold in AUC_0–t_ compared with the commercial product	[41]
Simvastatin P407	Antisolvent precipitation	137.8	Drug suspension	Increase of 6 h in T_max_, 73 h in MRT, and a decrease by 2-fold in C_max_	[42]
Rebamipide P188 + P407	Antisolvent precipitation	148.0 ± 2.5	Commercial product	No significant difference between the tablets	[43]
Gliclazide SDS + lecithin	Antisolvent precipitation	96.5 ± 15.0	Drug suspended in CMC Commercial product	Increase by 3-fold in C_max_ and 1.7-fold in AUC_0–t_ compared with the commercial product	[44]
Cyclosporin A HPC + TPGS + SDS	WBM	280–522	Commercial microemulsion suspended in water	Smaller nanocrystals increased C_max_ and AUC_0–48h_ compared with larger ones	[45]
Isoliquiritigenin mPEG-PCL	Antisolvent precipitation	46–540.0	Active compound suspension	Nanocrystals of ~300 nm increased 5.8-fold of C_max_ and 2.7-fold in AUC_0–t_	[46]
Nilotinib Soluplus^®^ + HPMCAS	Antisolvent precipitation	130.5 ± 1.2	Drug suspension in MC	Increase by 1.5-fold in C_max_ and 1.5-fold in AUC_0–24h_	[47]
Dolutegravir T80 + T20 + Soluplus^®^	HSH–antisolvent precipitation	337.1 ± 0.2	Drug suspension in MC	Increase by 3-fold in C_max_, 1.9-fold in AUC, and prolonged t_1/2_	[48]
Mangiferin HPMC + P407 + T80	HSH–antisolvent precipitation	100.2	Active compound suspension	Increase by 1.1-fold in C_max_, 1.2-fold in AUC_0–t_, prolonged t_1/2_, and MRT	[49]
Entrectinib P188	Antisolvent precipitation	83 ± 15	Drug suspension in HPMC Vehicle	Increase by 4-fold in C_max_ and 3-fold in AUC_0–24h_; elimination of food effect	[50]
Quercetin HPMC E15, P188, P407, TPGS or GL	WBM	193–210	Active compound suspended in water	Almost 11-fold increase in AUC_0–t_ for HPMC E15 and P188 preparations	[51]

HPMC: Hydroxypropyl methylcellulose; SDS: sodium dodecyl sulfate; P188: poloxamer 188; WBW: wet bead milling; HPH: high-pressure homogenization; HSH: high-speed homogenization; PVA: polyvinyl alcohol; Na-CMC: sodium carboxymethyl cellulose; T80: Tween 80; HPCS: hydroxypropyl chitosan; P407: poloxamer 407; CMC: carboxymethyl cellulose; HPC: hydroxy methylcellulose; TPGS: D-tocopheryl polyethylene glycol succinate; mPEG-PCL: methoxyl polyethylene glycol-polycaprolactone; HPMCAS: hydroxypropyl methylcellulose acetate succinate; MC: methyl cellulose; T20: Tween 20; GL: glycyrrhizin acid. Soluplus® and Cremophor® were introduced by BASF (Ludwigshafen, Germany).

## References

[1] Alqahtani M.S., Kazi M., Alsenaidy M.A., Ahmad M.Z. (2021). Advances in Oral Drug Delivery. Front. Pharmacol..

[2] van der Merwe J., Steenekamp J., Steyn D., Hamman J. (2020). The Role of Functional Excipients in Solid Oral Dosage Forms to Overcome Poor Drug Dissolution and Bioavailability. Pharmaceutics.

[3] Deng F., Bae Y.H. (2020). Bile Acid Transporter-Mediated Oral Drug Delivery. J. Control. Release.

[4] Dahan A., Hoffman A. (2008). Rationalizing the Selection of Oral Lipid Based Drug Delivery Systems by an in Vitro Dynamic Lipolysis Model for Improved Oral Bioavailability of Poorly Water Soluble Drugs. J. Control. Release.

[5] Koziolek M., Grimm M., Schneider F., Jedamzik P., Sager M., Kühn J.-P., Siegmund W., Weitschies W. (2016). Navigating the Human Gastrointestinal Tract for Oral Drug Delivery: Uncharted Waters and New Frontiers. Adv. Drug Deliv. Rev..

[6] Cheng L., Wong H. (2020). Food Effects on Oral Drug Absorption: Application of Physiologically-Based Pharmacokinetic Modeling as a Predictive Tool. Pharmaceutics.

[7] O’Shea J.P., Holm R., O’Driscoll C.M., Griffin B.T. (2019). Food for Thought: Formulating Away the Food Effect—A PEARRL Review. J. Pharm. Pharmacol..

[8] Rangaraj N., Sampathi S., Junnuthula V., Kolimi P., Mandati P., Narala S., Nyavanandi D., Dyawanapelly S. (2022). Fast-Fed Variability: Insights into Drug Delivery, Molecular Manifestations, and Regulatory Aspects. Pharmaceutics.

[9] Liu L., Tian C., Dong B., Xia M., Cai Y., Hu R., Chu X. (2021). Models to Evaluate the Barrier Properties of Mucus during Drug Diffusion. Int. J. Pharm..

[10] Tian Z., Mai Y., Meng T., Ma S., Gou G., Yang J. (2021). Nanocrystals for Improving Oral Bioavailability of Drugs: Intestinal Transport Mechanisms and Influencing Factors. AAPS PharmSciTech.

[11] Sugano K. (2010). Possible Reduction of Effective Thickness of Intestinal Unstirred Water Layer by Particle Drifting Effect. Int. J. Pharm..

[12] Elmeliegy M., Vourvahis M., Guo C., Wang D.D. (2020). Effect of P-Glycoprotein (P-Gp) Inducers on Exposure of P-Gp Substrates: Review of Clinical Drug–Drug Interaction Studies. Clin. Pharmacokinet..

[13] Lin J.H., Yamazaki M. (2003). Role of P-Glycoprotein in Pharmacokinetics. Clin. Pharmacokinet..

[14] Sanches B.M.A., Ferreira E.I. (2019). Is Prodrug Design an Approach to Increase Water Solubility?. Int. J. Pharm..

[15] Patel M., Hirlekar R. (2019). Multicomponent Cyclodextrin System for Improvement of Solubility and Dissolution Rate of Poorly Water Soluble Drug. Asian J. Pharm. Sci..

[16] Sathisaran I., Dalvi S. (2018). Engineering Cocrystals of Poorly Water-Soluble Drugs to Enhance Dissolution in Aqueous Medium. Pharmaceutics.

[17] Karagianni A., Kachrimanis K., Nikolakakis I. (2018). Co-Amorphous Solid Dispersions for Solubility and Absorption Improvement of Drugs: Composition, Preparation, Characterization and Formulations for Oral Delivery. Pharmaceutics.

[18] Haneef J., Ali S., Chadha R. (2021). Emerging Multi-Drug Eutectics: Opportunities and Challenges. AAPS PharmSciTech.

[19] Mohammad I.S., Hu H., Yin L., He W. (2019). Drug Nanocrystals: Fabrication Methods and Promising Therapeutic Applications. Int. J. Pharm..

[20] McGuckin M.B., Wang J., Ghanma R., Qin N., Palma S.D., Donnelly R.F., Paredes A.J. (2022). Nanocrystals as a Master Key to Deliver Hydrophobic Drugs via Multiple Administration Routes. J. Control. Release.

[21] Bilgili E., Guner G. (2021). Mechanistic Modeling of Wet Stirred Media Milling for Production of Drug Nanosuspensions. AAPS PharmSciTech.

[22] Soni G., Kale K., Shetty S., Gupta M.K., Yadav K.S. (2020). Quality by Design (QbD) Approach in Processing Polymeric Nanoparticles Loading Anticancer Drugs by High Pressure Homogenizer. Heliyon.

[23] Macedo L.d.O., Barbosa E.J., Löbenberg R., Bou-Chacra N.A. (2021). Anti-Inflammatory Drug Nanocrystals: State of Art and Regulatory Perspective. Eur. J. Pharm. Sci..

[24] Chary P.S., Shaikh S., Bhavana V., Rajana N., Vasave R., Mehra N.K. (2024). Emerging Role of Nanocrystals in Pharmaceutical Applications: A Review of Regulatory Aspects and Drug Development Process. Appl. Mater. Today.

[25] Chen Z., Wu W., Lu Y. (2020). What Is the Future for Nanocrystal-Based Drug-Delivery Systems?. Ther. Deliv..

[26] Li J., Wang Z., Zhang H., Gao J., Zheng A. (2021). Progress in the Development of Stabilization Strategies for Nanocrystal Preparations. Drug Deliv..

[27] Arti S., Bharti M., Kumar V., Saruchi, Rehani V., Dhiman J., Mallakpour S., Hussain C.M. (2022). Drug Nanocrystals as Nanocarrier-Based Drug Delivery Systems. Industrial Applications of Nanocrystals.

[28] Joseph E., Singhvi G., Grumezescu A.M. (2019). Multifunctional Nanocrystals for Cancer Therapy: A Potential Nanocarrier. Nanomaterials for Drug Delivery and Therapy.

[29] Chiou W.L., Jeong H.Y., Chung S.M., Wu T.C. (2000). Evaluation of Using Dog as an Animal Model to Study the Fraction of Oral Dose Absorbed of 43 Drugs in Humans. Pharm. Res..

[30] Arndt M., Chokshi H., Tang K., Parrott N.J., Reppas C., Dressman J.B. (2013). Dissolution Media Simulating the Proximal Canine Gastrointestinal Tract in the Fasted State. Eur. J. Pharm. Biopharm..

[31] Martinez M.N., Papich M.G., Fahmy R. (2022). Impact of Gastrointestinal Differences in Veterinary Species on the Oral Drug Solubility, in Vivo Dissolution, and Formulation of Veterinary Therapeutics. ADMET DMPK.

[32] Papich M.G., Martinez M.N. (2015). Applying Biopharmaceutical Classification System (BCS) Criteria to Predict Oral Absorption of Drugs in Dogs: Challenges and Pitfalls. AAPS J..

[33] Xu X., Chen G., Li Y., Wang J., Yin J., Ren L. (2019). Enhanced Dissolution and Oral Bioavailability of Cinacalcet Hydrochlorde Nanocrystals with No Food Effect. Nanotechnology.

[34] Karakucuk A., Teksin Z.S., Eroglu H., Celebi N. (2019). Evaluation of Improved Oral Bioavailability of Ritonavir Nanosuspension. Eur. J. Pharm. Sci..

[35] Guo M., Wei M., Li W., Guo M., Guo C., Ma M., Wang Y., Yang Z., Li M., Fu Q. (2019). Impacts of Particle Shapes on the Oral Delivery of Drug Nanocrystals: Mucus Permeation, Transepithelial Transport and Bioavailability. J. Control. Release.

[36] Liu Y., Liu W., Xiong S., Luo J., Li Y., Zhao Y., Wang Q., Zhang Z., Chen X., Chen T. (2020). Highly Stabilized Nanocrystals Delivering Ginkgolide B in Protecting against the Parkinson’s Disease. Int. J. Pharm..

[37] Imono M., Uchiyama H., Yoshida S., Miyazaki S., Tamura N., Tsutsumimoto H., Kadota K., Tozuka Y. (2020). The Elucidation of Key Factors for Oral Absorption Enhancement of Nanocrystal Formulations: In Vitro—In Vivo Correlation of Nanocrystals. Eur. J. Pharm. Biopharm..

[38] Kala S.G., Chinni S. (2021). Development and Characterization of Venetoclax Nanocrystals for Oral Bioavailability Enhancement. AAPS PharmSciTech.

[39] Zhu Y., Fu Y., Zhang A., Wang X., Zhao Z., Zhang Y., Yin T., Gou J., Wang Y., He H. (2022). Rod-Shaped Nintedanib Nanocrystals Improved Oral Bioavailability through Multiple Intestinal Absorption Pathways. Eur. J. Pharm. Sci..

[40] Shaikh F., Patel M., Patel V., Patel A., Shinde G., Shelke S., Pathan I. (2022). Formulation and Optimization of Cilnidipine Loaded Nanosuspension for the Enhancement of Solubility, Dissolution and Bioavailability. J. Drug Deliv. Sci. Technol..

[41] Liu J., Li S., Ao W., Li Y., Xiao Y., Bai M. (2022). Fabrication of an Aprepitant Nanosuspension Using Hydroxypropyl Chitosan to Increase the Bioavailability. Biochem. Biophys. Res. Commun..

[42] Bakhaidar R.B., Naveen N.R., Basim P., Murshid S.S., Kurakula M., Alamoudi A.J., Bukhary D.M., Jali A.M., Majrashi M.A., Alshehri S. (2022). Response Surface Methodology (RSM) Powered Formulation Development, Optimization and Evaluation of Thiolated Based Mucoadhesive Nanocrystals for Local Delivery of Simvastatin. Polymers.

[43] Jin G., Ngo H.V., Wang J., Cui J.-H., Cao Q.-R., Park C., Jung M., Lee B.-J. (2022). Design and Evaluation of in Vivo Bioavailability in Beagle Dogs of Bilayer Tablet Consisting of Immediate Release Nanosuspension and Sustained Release Layers of Rebamipide. Int. J. Pharm..

[44] Sampathi S., Prajapati S., Junnuthula V., Dyawanapelly S. (2022). Pharmacokinetics and Anti-Diabetic Studies of Gliclazide Nanosuspension. Pharmaceutics.

[45] Sun W., Gao J., Fan R., Zhang T., Tian Y., Wang Z., Zhang H., Zheng A. (2022). The Effect of Particle Size on the Absorption of Cyclosporin A Nanosuspensions. Int. J. Nanomed..

[46] Ma Y., Yang X., Chen G., Zhang Y., Zhang H., Zhang W. (2022). Effect of Particle Size on the Oral Absorption of Isoliquiritigenin Nanocrystals. Braz. J. Pharm. Sci..

[47] Chougule M., Sirvi A., Saini V., Kashyap M., Sangamwar A.T. (2023). Enhanced Biopharmaceutical Performance of Brick Dust Molecule Nilotinib via Stabilized Amorphous Nanosuspension Using a Facile Acid–Base Neutralization Approach. Drug Deliv. Transl. Res..

[48] Bhairam M., Pandey R.K., Shukla S.S., Gidwani B. (2023). Preparation, Optimization, and Evaluation of Dolutegravir Nanosuspension: In Vitro and In Vivo Characterization. J. Pharm. Innov..

[49] Sarwar A.R., Iqbal F.M., Jamil M.A., Abbas K. (2023). Nanocrystals of Mangiferin Using Design Expert: Preparation, Characterization, and Pharmacokinetic Evaluation. Molecules.

[50] Chary S.S., Bhikshapathi D.V.R.N., Vamsi N.M., Kumar J.P. (2024). Optimizing Entrectinib Nanosuspension: Quality by Design for Enhanced Oral Bioavailability and Minimized Fast-Fed Variability. Bionanoscience.

[51] Zhu Y., Hu F., Shen C., Shen B., Yuan H. (2024). Quercetin Nanocrystals for Bioavailability Enhancement: Impact of Different Functional Stabilizers on In Vitro/In Vivo Drug Performances. Pharm. Dev. Technol..

[52] Malamatari M., Taylor K.M.G., Malamataris S., Douroumis D., Kachrimanis K. (2018). Pharmaceutical Nanocrystals: Production by Wet Milling and Applications. Drug Discov. Today.

[53] Vinchhi P., Patel J.K., Patel M.M., Patel J.K., Pathak Y.V. (2021). High-Pressure Homogenization Techniques for Nanoparticles. Emerging Technologies for Nanoparticle Manufacturing.

[54] Sinha B., Müller R.H., Möschwitzer J.P. (2013). Bottom-up Approaches for Preparing Drug Nanocrystals: Formulations and Factors Affecting Particle Size. Int. J. Pharm..

[55] Rabanal-Ruiz Y., Llanos-González E., Alcain F.J. (2021). The Use of Coenzyme Q10 in Cardiovascular Diseases. Antioxidants.

[56] Sun J., Wang F., Sui Y., She Z., Zhai W., Wang C., Deng Y. (2012). Effect of Particle Size on Solubility, Dissolution Rate, and Oral Bioavailability: Evaluation Using Coenzyme Q10 as Naked Nanocrystals. Int. J. Nanomed..

[57] Uhlemann J., Diedam H., Hoheisel W., Schikarski T., Peukert W. (2020). Modeling and Simulation of Process Technology for Nanoparticulate Drug Formulations—A Particle Technology Perspective. Pharmaceutics.

[58] Miller C.C. (1924). The Stokes-Einstein Law for Diffusion in Solution. Proc. R. Soc. Lond. A.

[59] Yu M., Wang J., Yang Y., Zhu C., Su Q., Guo S., Sun J., Gan Y., Shi X., Gao H. (2016). Rotation-Facilitated Rapid Transport of Nanorods in Mucosal Tissues. Nano Lett..

[60] Noyes A.A., Whitney W.R. (1897). The Rate of Solution of Solid Substances in Their Own Solutions. J. Am. Chem. Soc..

[61] Junyaprasert V.B., Morakul B. (2015). Nanocrystals for Enhancement of Oral Bioavailability of Poorly Water-Soluble Drugs. Asian J. Pharm. Sci..

[62] Gao L., Liu G., Ma J., Wang X., Zhou L., Li X., Wang F. (2013). Application of Drug Nanocrystal Technologies on Oral Drug Delivery of Poorly Soluble Drugs. Pharm. Res..

[63] Rangaraj N., Pailla S.R., Chowta P., Sampathi S. (2019). Fabrication of Ibrutinib Nanosuspension by Quality by Design Approach: Intended for Enhanced Oral Bioavailability and Diminished Fast Fed Variability. AAPS PharmSciTech.

[64] Mou D., Chen H., Wan J., Xu H., Yang X. (2011). Potent Dried Drug Nanosuspensions for Oral Bioavailability Enhancement of Poorly Soluble Drugs with PH-Dependent Solubility. Int. J. Pharm..

[65] Thombre A.G., Caldwell W.B., Friesen D.T., McCray S.B., Sutton S.C. (2012). Solid Nanocrystalline Dispersions of Ziprasidone with Enhanced Bioavailability in the Fasted State. Mol. Pharm..

[66] Reddy M.R., Gubbiyappa K.S. (2023). Formulation Development, Optimization, and Characterization of Entrectinib-Loaded Supersaturable Self-Nanoemulsifying Drug Delivery Systems. Bionanoscience.

[67] Feldman M., Barnett C. (1991). Fasting Gastric PH and Its Relationship to True Hypochlorhydria in Humans. Dig. Dis. Sci..

[68] Gao L., Liu G., Ma J., Wang X., Zhou L., Li X. (2012). Drug Nanocrystals: In Vivo Performances. J. Control. Release.

[69] Food and Drug Administration (FDA) Chemistry Review: Norvir (Ritonavir) Tablets. https://www.accessdata.fda.gov/drugsatfda_docs/nda/2010/022417s000_ChemR.pdf.

[70] Food and Drug Administration (FDA) Product Quality Review: Entrectinib Capsules. https://www.accessdata.fda.gov/drugsatfda_docs/nda/2019/212725Orig1s000,%20212726Orig1s000ChemR.pdf.

[71] Wang Y., Tan X., Fan X., Zhao L., Wang S., He H., Yin T., Zhang Y., Tang X., Jian L. (2021). Current Strategies for Oral Delivery of BCS IV Drug Nanocrystals: Challenges, Solutions and Future Trends. Expert. Opin. Drug Deliv..

[72] Arora S., Pansari A., Kilford P., Jamei M., Gardner I., Turner D.B. (2020). Biopharmaceutic In Vitro In Vivo Extrapolation (IVIV_E) Informed Physiologically-Based Pharmacokinetic Model of Ritonavir Norvir Tablet Absorption in Humans Under Fasted and Fed State Conditions. Mol. Pharm..

[73] Ng J., Klein C., Chui Y., Awni W., Morris J., Podsadecki T., Cui Y., Bernstein B., Kim D. (2008). The Effect of Food on Ritonavir Bioavailability Following Administration of Ritonavir 100 Mg Film-Coated Tablet in Healthy Adult Subjects. J. Int. AIDS Soc..

[74] Vermunt M.A.C., de Weger V.A., Janssen J.M., Lopez-Yurda M.I., Keessen M., Thijssen B., Rosing H., Huitema A.D.R., Beijnen J.H., Marchetti S. (2021). Effect of Food on the Pharmacokinetics of the Oral. Docetaxel Tablet Formulation ModraDoc006 Combined with Ritonavir (ModraDoc006/r) in Patients with Advanced Solid Tumours. Drugs R&D.

[75] DeSesso J.M., Jacobson C.F. (2001). Anatomical and Physiological Parameters Affecting Gastrointestinal Absorption in Humans and Rats. Food Chem. Toxicol..

[76] Cao X., Gibbs S.T., Fang L., Miller H.A., Landowski C.P., Shin H.-C., Lennernas H., Zhong Y., Amidon G.L., Yu L.X. (2006). Why Is It Challenging to Predict Intestinal Drug Absorption and Oral Bioavailability in Human Using Rat Model. Pharm. Res..

[77] Femia R.A., Goyette R.E. (2005). The Science of Megestrol Acetate Delivery. BioDrugs.

[78] Park S.R., Hwang J.G., Jeong S.I., Choi Y.-S., Min H.J., Kim H.Y., Choi B.-H., Park M.K. (2024). Comparison of the Pharmacokinetic Characteristics and Bioequivalence between Two Nanosuspension Formulations of Megestrol Acetate in Healthy Korean Male Subjects. Transl. Clin. Pharmacol..

[79] Koziolek M., Grimm M., Bollmann T., Schäfer K.J., Blattner S.M., Lotz R., Boeck G., Weitschies W. (2019). Characterization of the GI Transit Conditions in Beagle Dogs with a Telemetric Motility Capsule. Eur. J. Pharm. Biopharm..

[80] Apley M., Crist B., Gonzalez M., Hunter R.P., Martinez M.N., Modric S., Papich M.G., Parr A.F., Riviere J.E., Marques M.R.C. (2013). Solubility Criteria for Veterinary Drugs—Workshop Report. Pharm. Forum.

[81] Jang K., Yoon S., Kim S.-E., Cho J.-Y., Yoon S.H., Lim K.S., Yu K.-S., Jang I.-J., Lee H. (2014). Novel Nanocrystal Formulation of Megestrol Acetate Has Improved Bioavailability Compared with the Conventional Micronized Formulation in the Fasting State. Drug Des. Devel Ther..

[82] Xiong S., Liu W., Li D., Chen X., Liu F., Yuan D., Pan H., Wang Q., Fang S., Chen T. (2019). Oral Delivery of Puerarin Nanocrystals To Improve Brain Accumulation and Anti-Parkinsonian Efficacy. Mol. Pharm..

[83] García-Herrero V., Torrado C., García-Rodríguez J.J., López-Sánchez A., Torrado S., Torrado-Santiago S. (2017). Improvement of the Surface Hydrophilic Properties of Naproxen Particles with Addition of Hydroxypropylmethyl Cellulose and Sodium Dodecyl Sulphate: In Vitro and in Vivo Studies. Int. J. Pharm..

[84] Narula A., Sabra R., Li N. (2022). Mechanisms and Extent of Enhanced Passive Permeation by Colloidal Drug Particles. Mol. Pharm..

[85] Ramachandran G., Sudheesh M.S. (2021). Role of Permeability on the Biopredictive Dissolution of Amorphous Solid Dispersions. AAPS PharmSciTech.

[86] Amidon G.L., Lennernäs H., Shah V.P., Crison J.R. (1995). A Theoretical Basis for a Biopharmaceutical Drug Classification: The Correlation of in Vitro Drug Product Dissolution and in Vivo Bioavailability. Pharm. Res..

[87] Amidon K.S., Langguth P., Lennernäs H., Yu L., Amidon G.L. (2011). Bioequivalence of Oral Products and the Biopharmaceutics Classification System: Science, Regulation, and Public Policy. Clin. Pharmacol. Ther..

[88] Gigliobianco M.R., Casadidio C., Censi R., Di Martino P. (2018). Nanocrystals of Poorly Soluble Drugs: Drug Bioavailability and Physicochemical Stability. Pharmaceutics.

[89] Yasir M., Asif M., Kumar A., Aggarval A. (2010). Biopharmaceutical Classification System: An Account. Int. J. Pharmtech Res..

[90] Peltonen L., Strachan C.J. (2020). Degrees of Order: A Comparison of Nanocrystal and Amorphous Solids for Poorly Soluble Drugs. Int. J. Pharm..

[91] Jermain S.V., Brough C., Williams R.O. (2018). Amorphous Solid Dispersions and Nanocrystal Technologies for Poorly Water-Soluble Drug Delivery—An Update. Int. J. Pharm..

[92] Merisko-Liversidge E., Liversidge G.G. (2011). Nanosizing for Oral and Parenteral Drug Delivery: A Perspective on Formulating Poorly-Water Soluble Compounds Using Wet Media Milling Technology. Adv. Drug Deliv. Rev..

[93] Tuomela A., Hirvonen J., Peltonen L. (2016). Stabilizing Agents for Drug Nanocrystals: Effect on Bioavailability. Pharmaceutics.

[94] Fu Q., Sun J., Ai X., Zhang P., Li M., Wang Y., Liu X., Sun Y., Sui X., Sun L. (2013). Nimodipine Nanocrystals for Oral Bioavailability Improvement: Role of Mesenteric Lymph Transport in the Oral Absorption. Int. J. Pharm..

[95] Yu Q., Wang Z., Li P., Yang Q. (2013). The Effect of Various Absorption Enhancers on Tight Junction in the Human Intestinal Caco-2 Cell Line. Drug Dev. Ind. Pharm..

[96] Brunner J., Ragupathy S., Borchard G. (2021). Target Specific Tight Junction Modulators. Adv. Drug Deliv. Rev..

[97] Luiz M.T., Di Filippo L.D., Alves R.C., Araújo V.H.S., Duarte J.L., Marchetti J.M., Chorilli M. (2021). The Use of TPGS in Drug Delivery Systems to Overcome Biological Barriers. Eur. Polym. J..

[98] Collnot E.-M., Baldes C., Schaefer U.F., Edgar K.J., Wempe M.F., Lehr C.-M. (2010). Vitamin E TPGS P-Glycoprotein Inhibition Mechanism: Influence on Conformational Flexibility, Intracellular ATP Levels, and Role of Time and Site of Access. Mol. Pharm..

[99] Macedo L.d.O., Morales I.A.C., Barbosa E.J., Stephano M.A., de Araujo G.L.B., Bou-Chacra N.A. (2022). Thermal Study, Process Optimization, and Water Solubility Improvement of a Freeze-Dried Artemether Nanosuspension for Malaria Treatment. J. Drug Deliv. Sci. Technol..

[100] Guo Y., Luo J., Tan S., Otieno B.O., Zhang Z. (2013). The Applications of Vitamin E TPGS in Drug Delivery. Eur. J. Pharm. Sci..

[101] Kou L., Sun R., Bhutia Y.D., Yao Q., Chen R. (2018). Emerging Advances in P-Glycoprotein Inhibitory Nanomaterials for Drug Delivery. Expert. Opin. Drug Deliv..

[102] Halder J., Pradhan D., Kar B., Ghosh G., Rath G. (2022). Nanotherapeutics Approaches to Overcome P-Glycoprotein-Mediated Multi-Drug Resistance in Cancer. Nanomedicine.

[103] Subramanian D.A., Langer R., Traverso G. (2022). Mucus Interaction to Improve Gastrointestinal Retention and Pharmacokinetics of Orally Administered Nano-Drug Delivery Systems. J. Nanobiotechnol..

[104] Cone R.A. (2009). Barrier Properties of Mucus. Adv. Drug Deliv. Rev..

[105] Feng C., Chen X., Zhang J., Sun G., Cheng X., Wang Z., Park H.-J. (2011). The Effect of Carboxymethyl-Chitosan Nanoparticles on Proliferation of Keloid Fibroblast. Front. Chem. China.

[106] Tan S.L.J., Billa N. (2021). Improved Bioavailability of Poorly Soluble Drugs through Gastrointestinal Muco-Adhesion of Lipid Nanoparticles. Pharmaceutics.

[107] Liu J., Tu L., Cheng M., Feng J., Jin Y. (2020). Mechanisms for Oral Absorption Enhancement of Drugs by Nanocrystals. J. Drug Deliv. Sci. Technol..

[108] Tian Z., Zhao Y., Mai Y., Qiao F., Guo J., Dong L., Niu Y., Gou G., Yang J. (2022). Nanocrystals with Different Stabilizers Overcome the Mucus and Epithelial Barriers for Oral Delivery of Multicomponent Bufadienolides. Int. J. Pharm..

[109] Sillman B., Bade A.N., Dash P.K., Bhargavan B., Kocher T., Mathews S., Su H., Kanmogne G.D., Poluektova L.Y., Gorantla S. (2018). Creation of a Long-Acting Nanoformulated Dolutegravir. Nat. Commun..

[110] Jahangir M.A., Imam S.S., Muheem A., Chettupalli A., Al-Abbasi F.A., Nadeem M.S., Kazmi I., Afzal M., Alshehri S. (2022). Nanocrystals: Characterization Overview, Applications in Drug Delivery, and Their Toxicity Concerns. J. Pharm. Innov..

[111] Ahmad A., Imran M., Sharma N. (2022). Precision Nanotoxicology in Drug Development: Current Trends and Challenges in Safety and Toxicity Implications of Customized Multifunctional Nanocarriers for Drug-Delivery Applications. Pharmaceutics.

[112] Seok S.H., Ha J.-M., Kim T.H., Kim G.-W., Kim B.H., Lee D.W., Choi M.-S., Lee S.-H., Kim J.-Y., Park E.-S. (2021). Effect of Gastric Residence Time on the Oral Absorption of Rebamipide Sustained-Release Tablets in Beagle Dogs. J. Pharm. Investig..

[113] Nguyen T.-T., Hwang K.-M., Kim S.-H., Park E.-S. (2020). Development of Novel Bilayer Gastroretentive Tablets Based on Hydrophobic Polymers. Int. J. Pharm..

[114] Lv Y., Wu W., Corpstein C.D., Li T., Lu Y. (2022). Biological and Intracellular Fates of Drug Nanocrystals through Different Delivery Routes: Recent Development Enabled by Bioimaging and PK Modeling. Adv. Drug Deliv. Rev..

[115] Nowacek A.S., McMillan J., Miller R., Anderson A., Rabinow B., Gendelman H.E. (2010). Nanoformulated Antiretroviral Drug Combinations Extend Drug Release and Antiretroviral Responses in HIV-1-Infected Macrophages: Implications for NeuroAIDS Therapeutics. J. Neuroimmune Pharmacol..

